# A mixed methods study of schema modes amongst people living with eating disorders

**DOI:** 10.1186/s40337-024-01031-x

**Published:** 2024-06-12

**Authors:** Clare Marney, Marie Reid, Bernice Wright

**Affiliations:** School of Psychology and Social Work, University of Hull, Hull, UK

**Keywords:** Eating disorders, Schema therapy, Schema modes, Trauma, Adverse childhood experiences, Control, Thematic analysis, Qualitative

## Abstract

**Background:**

Schema therapy is promising for people with eating disorders, especially those unresponsive to cognitive behavioural therapy. Complex underlying psychological constructs include dysfunctional schemas and maladaptive modes. This study aimed to explore people living with eating disorders’ schema modes and their identification with and understanding of their high scoring modes.

**Methods:**

Sixteen women with enduring eating disorders without prior exposure to schema therapy completed the schema mode inventory for eating disorders short form (SMI-ED-SF), then participated in semi-structured interviews discussing their high scoring modes. Interviews were analysed by thematic analysis.

**Results:**

All participants scored above clinical concern on at least one maladaptive mode and many scored high on multiple modes, most commonly Demanding Mode, Vulnerable Child and Detached Self-Soother. Qualitatively, four themes emerged: 1) Adverse family environments related to (a) trauma and the vulnerable and angry child and (b) unrealistically high standards; 2) Mode effects on (a) everyday life and (b) disordered eating; 3) Modes are psychologically protective in (a) avoiding emotion by detachment and soothing, (b) people pleasing by compliance and surrender; 4) Help seeking including (a) barriers to recovery from an eating disorder, (b) dissatisfaction with interventions experienced to date, (c) schema therapy as a promising alternative.

**Discussion:**

Participants recognised and identified with their high scoring schema modes. After negative experiences with previous interventions, they considered schema therapy to be a promising alternative that could understand and work on their deeper psychological issues. This suggests that schema modes are a promising way of understanding and working with enduring eating disorders.

## Abstract (plain language summary)

Maladaptive modes are important in Schema Therapy for eating disorders. They are momentary patterns of thought, feeling and behaviour, triggered by experiences to which people with eating disorders can be oversensitive. In a child mode the person may be inexplicably childish. A coping mode may involve detachment, surrender to others, or perfectionist overcompensation. A parent mode may involve unrealistic standards and demands. There are also two healthy modes of Healthy Adult and Happy Child, which are often lacking in people with eating disorders. In this study women with enduring eating disorders completed the schema mode inventory and then discussed their high scoring modes. Common maladaptive modes were vulnerable child, demanding parent mode and perfectionistic over-compensator. Participants identified with their high scoring modes and thought them useful for self-understanding, so schema therapy is a promising way of understanding the psychopathology underlying enduring eating disorders.

## Background

Enhanced cognitive behavioural therapy (CBT-E) is recommended for eating disorders (ED) [1], but about half of those treated are non-responsive [2–4]. Anorexia Nervosa is additionally often hindered by ambivalence about the disorder, resistance to CBT-E techniques, significant drop-out rates [5] and high levels of relapse [6].

Further complexities include co-morbidity with anxiety or affective disorders [6, 7], low self-esteem, and stressful work and social situations [8]. Moreover, 58–69% of individuals living with ED meet diagnostic criteria for a personality disorder [9] and rigid personality traits such as perfectionism, neuroticism, or avoidance motivation are also common [10]. Finally, the family environment, including any adverse experiences such as trauma, abuse and neglect, influence development of EDs and personality disorders [11], as can experiencing overindulgence and over protection as a child [2, 12–14]. Additional interventions such as schema therapy that better address the complexities around the disorder may be useful [2, 15].

Schema therapy is an integrative therapy and an alternative to CBT/CBT-E [2, 15, 16]. It has been effective with other difficult to treat populations [2, 17] and has a strong focus on the therapeutic relationship. It explores childhood and adolescent experiences to address the origins of underlying psychological issues [17]. It may therefore be particularly beneficial to people living with ED, who have disproportionate levels of adverse childhood experiences compared to controls [11] and the severity of adversity is related to severity of symptoms and negative self-image [18].

Schema therapy theorises that EDs are manifestations of deeper psychological functions, encapsulated as schemas and modes [9]. Common schemas in EDs include unrelenting standards, defectiveness/shame, social isolation and social undesirability [16, 19]. Clients’ entrenched and difficult to change cognitions, feelings and behaviours [2, 17] are caused by maladaptive schemas that are usually learned in childhood as ways of coping with, or responses to, difficulties in childhood. Such difficulties can be due to maltreatment, neglect or abuse, or simply to a poor fit between the child’s psychological needs and their psychosocial environment. For example, a highly sensitive child may form a defectiveness/shame schema in reaction to criticism that a more resilient child would ignore.

Maladaptive schemas strongly resist change and perpetuate problematically in adulthood, applied to situations where they are not appropriate. They are patterns of cognitions, feelings and bodily sensations, but are not themselves behaviour. People react to a maladaptive schema being activated either by surrendering to it, avoiding it, or over-compensating for it [17]. For example, unrelenting standards may lead to setting unachievably high standards, or avoiding tasks that are challenging for the person, or failing at demanding tasks to demonstrate that high standards are unimportant.

However, schema therapy for eating disorders has developed to focus on maladaptive modes, the focus of this research. Modes are repeating patterns of cognitions, feelings, and behaviours where underlying maladaptive schemas and reactions to those schemas are manifest. More than one schema at a time, plus their reactions to them, can manifest in a mode.

People with extreme characterological issues, the preferred term to personality disorders [17], can visibly manifest different modes during therapy, and in everyday life sometimes by switching their tone of voice, facial expression and conversational content. For example, clients with EDs when in compliant surrenderer mode may present as agreeable to and compliant with therapeutic requirements, such as weight gain, until they are actually expected to gain weight, when they may switch to detached protector mode. This change can be visible for compliant surrender is pleasant and agreeable, while detached protector is disagreeable and distant. People with less extreme issues may manifest modes more subtly. It is theorised that modes are idiosyncratic; not everyone has all modes and the nature and naming of modes is individual within therapy. However, research questionnaires use generalised modes, of which there are 16.

In quantitative studies, usually using the Schema Mode Inventory for Eating Disorders Short Form [20], compared to controls, people living with ED score higher on all modes other than bully and attack, and self-aggrandiser [16, 21]. The ED mode model includes the specific mode of eating disorders over-controller, focussed on eating, exercise and bodily appearance [9].

Another common and important mode in ED is the vulnerable child, which is the person’s fundamental unmet childhood needs of love, safety, being valued, spontaneity and play, and learning boundaries [22, 23]. People with ED often lack awareness of the vulnerable child, which is masked under critic or coping modes, commonly critical parent, detached protector [15] compliant surrenderer, perfectionist overcontroller and eating disorders over-controller.

This aligns with the theory that EDs develop when the child’s temperament (e.g. compulsive, perfectionistic, or emotionally sensitive) misaligns with their environment (e.g. abusive, critical, or emotionally disconnected) [24]. Indeed, schemas and modes mediate relationships between traumatic experiences in childhood and ED symptoms [25]. However, quantitative research does not elucidate how people experience their schemas and modes.

One qualitative study found that clients receiving schema therapy reported improved self-awareness, more ability to manage emotions, and recognition of the importance of exploring the origins of personal difficulties and working out how to navigate these. Participants preferred schema therapy’s focus on idiosyncratic and long-standing psychological factors [26]. One interpretative phenomenological case study [22, 23] explored how a client with anorexia nervosa experienced her modes during recorded schema therapy sessions. She described thoughts and behaviours attributable to vulnerable, angry, and healthy child modes, as well as to dysfunctional parent modes, although she did not experience punitive and demanding parent modes separately. Also prominent was the eating disorder overcontroller mode. It was concluded that the mode system related to her experience, but that a person’s modes and how they relate to each other are idiosyncratic. In another study, clients valued an individualistic ST approach, perceived staff as supportive, found treatment emotionally supportive and personalised, and reported high levels of satisfaction [27].

A clinically important aspect of ST is mode sequences [2] where first a schema is activated, triggering a mode, then that mode triggers another mode. For example, interpersonal conflict triggers vulnerable child mode, which in turn triggers detached protector or punitive critic modes. Both these can lead to ED behaviours. In detached protector, people may use bingeing to comfort themselves, or excessive restraint to block out negative thoughts. In punitive critic mode, people may use restraint to exert control over eating and/or exercise to punish themselves, or to demonstrate that they can control this part of life. In turn these modes can trigger eating disorders overcontroller.

Further qualitative research would be informative, particularly in the case of schema modes, to increase understanding of how ED pathology develops and is maintained and experienced by the person [2]. The present study explored how people with enduring eating disorders who had not been exposed to schema therapy felt about their maladaptive modes. Because modes are idiosyncratic, no specific predictions were made about which modes participants would have experienced, their origins, or how they impacted the person’s life.

## Methods

### Aim

To survey the schema mode scores of people living with enduring eating disorders, then explore whether they recognised, identified with, understood the development of, and considered how their high-scoring modes related to their disordered eating symptoms and behaviours.

### Design

In this mixed-methods study, volunteers with enduring eating disorders who had no prior exposure to schema therapy completed the schema mode inventory for eating disorders short form (SMI-ED-SF [20] then discussed their schema mode scores in a qualitative interview that was analysed with thematic analysis. Ethical approval for the study was granted by the University of Hull Faculty of Health Sciences, in accordance with the declaration of Helsinki.

### Participants

Participant characteristics are summarised in Table 1. Sixteen women (aged 22 to 65 years old) were recruited from an eating disorder support charity. This charity provides a range of support including a limited amount of evidence-based psychological therapy for eating disorders, group work, support from mentors and support by correspondence. In general, clients of the charity have had contact, sometime multiple contacts, with specialist eating disorder services but, for complex reasons to be discussed, often without completing any intervention. The study was advertised via the charity’s client list and participants volunteered with written informed consent, verified verbally at the start of interview.

**Table 1:** Participant information

Participant ID	Age Band	Occupation Sector	Highest Education Level	Disordered Eating Type	BMI	Duration	Diagnoses	Comorbidity	Psychological Therapy Received
1	18–30	Health & Social Care	MSc	AN	15.5	12 years	Mental Health Services	Self-harm	In-patient, CAT
2	31–40	Civil Service	A Level	BED	43.5	20 years	Self-diagnosed	Anxiety and Depression	CBT – (for anxiety and depression) Grief counselling
3	41–50	Health & Social Care	NNEB, NVQ2	AN	Weight not disclosed	20 years	GP & Mental Health Services		Day patient at ED services & CBT
4	51–60	Education	BSc Hons	AN	17.6	5 years	GP & Mental Health Services		Psychiatrist, psychologist, CBT
5	61–70	Retired	Level 3	BED	40.3	30 Years	GP	Anxiety and Depression	CBT (for anxiety and depression) & private psychotherapy
6	31–40	Health & Social Care	Undergraduate Degree	BN	19.2	19 Years	GP & Mental Health Services	Anxiety and Depression	Psychotherapy, EMDR, CBT
7	18–30	Education	Degree	AN	20.7	8 years	GP		Inpatient, CAMHS, CBT, private psychotherapy
8	51–60	Education	Diploma	BED	33.5	2 years	Mental Health Services		None but has attended CBT via MIND
9	31–40	HR	Post Graduate	AN	17.1	20 years	GP	Anxiety and Depression	ED specialists Private therapy CBT (for anxiety and depression)
10	51–60	Domestic Services	PGCE	AN	20.7	12 years	GP/Crisis Team	BPD, Anxiety and Depression and Asperger’s	DBT (for BPD), CAT, CBT, MBT
11	18–30	Administration	BSc	BED	29.8	10 years	GP		CBT (for anxiety and depression)
12	51–60	Legal	Degree	BN	26.2	16 years	GP	Anxiety and Depression, Bipolar 2	CBT
13	18–30	Education	A Levels	AN	20.5	4 years	GP/Consultant	Anxiety and Depression	Counselling
14	51–60	Service Industry	College	BED	Weight not disclosed	40 years	Self-diagnosed		Counselling/CBT
15	18–30	Administration	College	AN	17.8	4 years	Crisis Team		Psychologist (Crisis Team)
16	31–40	Service Industry	MSc	AN	19.3	26 years	GP		Psychologist

***Note: Abbreviation key -*** BPD, Borderline Personality Disorder; CAMHS, Child and Adolescent Mental Health Services; CAT, Cognitive Analytic Therapy; CBT, Cognitive Behaviour Therapy; DBT, Dialectical Behaviour Therapy; EMDR, Eye Movement Desensitisation and Reprocessing; MBT, Mentalisation Based Therapy

Nine participants identified as living with anorexia nervosa, five with binge eating disorder and two with bulimia nervosa. None identified with “other feeding or eating disorder”. All but two participants had received a diagnosis from either their GP or mental health services. Duration of ED’s ranged between two and forty years and 12/16 women reported that their disordered eating had lasted 10 years or more. The body mass index of the sample ranged from 15.46 to 43.53. Eight participants reported co-morbidities, some multiple, most commonly anxiety and depression.

All participants said that they had previously received some form of psychological intervention. Although not a focus of interviews, some reported difficulties accessing specialist eating disorder services due to eligibility issues, such as being of insufficiently low body mass, other issues such as scarcity of places, and difficulties engaging with the service.

Importantly, participants had not been previously exposed to the concepts of schema therapy. The charity has clients from all over the UK and so participants came from a wide geographical area. All participants were referred to the charity’s support if they found the research distressing.

### Measures

The Schema Mode Inventory Short Form [20] is a validated measure with good internal consistency. It measures 16 schema modes specific to disordered eating with 64 items (4 per mode) using a 6-point Likert scale (0 = never or almost never to 5 = always). The maladaptive modes are: Vulnerable Child; Angry Child; Enraged Child; Impulsive Child; Undisciplined Child; Punitive Mode; Demanding Mode; Compliant Surrenderer; Detached Protector; Detached Self-Soother; Self-Aggrandiser; Bully and Attack; Helpless Surrenderer; Eating Disorder Overcontroller. Two healthy modes are: Happy Child; Healthy Adult. Mean mode scores are calculated. A mean score of three or above is considered clinically significant [20]. The 16 modes are derived from clinical experience and questionnaire research to encompass the full spectrum of how people with eating disorders can be maladaptive. It is not expected that most people will score highly on every mode.

### Procedure

Participants completed the SMI-ED-SF and their clinically significant modes were scored. Then participants engaged in a semi-structured interview to explore their high-scoring modes in relation to their lived experiences and how this could relate to their disordered eating patterns. Interviews were conducted and recorded via the online platform Zoom. Interview duration varied from 45 to 90 min.

### Interviews

Each interview began with a brief overview of schema therapy and explanation of schema modes, to aid participants’ understanding of the questionnaire and their results. They were then asked a range of open-ended questions based on their life experiences in relation to modes. For example, *You scored high on X mode. Does this seem accurate for you? Are you aware of this mode? Do you have any idea about how X mode may have developed? What are the triggers for this mode in your life? How does it impact on your life and eating?* To get a clearer picture of how participants understood the modes they were asked *Can you relate to the mode label, or would you call it something else? Were you surprised/unsurprised by any of the modes? Are you aware of any efforts you make to challenge/change the mode?* To gain insight into their experience of discussing schema modes the interview concluded by asking *Hearing yourself vocalise all of this how do you feel now? Do you think you would find Schema Therapy beneficial?*

### Qualitative analysis

Interviews were transcribed verbatim by the first author with identifying information removed or redacted. Participants are referred to by code number. Transcripts were analysed using thematic analysis [28], following the phases of familiarising the self with the data; generation of initial codes; searching for and reviewing of themes; continued analysis to refine and identify main themes and subthemes and selection of illustrative extracts to accompany the themes in relation to the aims of the research. Extracts from the interviews used have been edited for brevity.

## Results

### Quantitative results

As can be seen in Table 2, only a minority of this cohort scored highly (3+) on the functional modes of Healthy Adult and Happy Child and all but two participants had more high scores on maladaptive than functional modes. All participants scored highly on at least one maladaptive mode and many scored highly on multiple modes with a range of between two and thirteen modes. The most common clinically significant modes were Demanding Mode, Vulnerable Child and Detached Self-Soother. Other coping modes, including Eating Disorder Overcontroller, Detached Protector, Compliant Surrenderer and Helpless Surrenderer, were clinically significant for at least half the sample. Other modes were identified by fewer participants. Enraged Child was identified by nobody and Impulsive Child and Bully and Attack by very few.

**Table 2:** Clinically significant participant modes with mean mode scores of 3+

		Positive modes		Child Modes					Dysfunctional Parent Modes		Maladaptive Coping Modes						Eating Disorder specific Mode
ID	ED type	Happy Child	Healthy Adult	Vulnerable	Angry	Enraged	Impulsive	Undisciplined	Punitive	Demanding	Compliant Surrenderer	Helpless Surrenderer	Detached Protector	Detached Self-Soother	Self Aggrandiser	Bully & Attack	ED Over-controller
15	AN			√	√					√	√	√	√	√		√	√
3	AN			√				√	√		√	√	√	√			
7	AN		√							√	√	√		√			√
10	AN			√						√	√	√	√	√			
4	AN			√						√	√	√	√	√			
9	AN	√	√							√							√
1	AN-BP		√	√	√					√		√	√	√	√		√
11	BED		√	√	√		√			√	√	√	√	√			√
8	BED			√	√						√			√			√
2	BED			√	√						√			√			
14	BED		√							√				√			
5	BED	√	√														
6	BN	√		√	√				√	√	√	√	√	√	√	√	√
12	BN	√	√							√							
16	Restricted			√						√			√				√
13	Unspecified	√								√		√	√	√	√		
*n* = 16	TOTAL	5	7	10	6	0	1	1	2	12	9	9	9	12	3	2	8

### Qualitative results

Thematic analysis of discussion of individual modes identified four themes: 1. Adverse family environments related to (a) trauma and the vulnerable and angry child and (b) unrealistically high standards; 2. Mode effects on (a) everyday life and (b) disordered eating; 3. Modes have protective functions in (a) avoiding emotion by detachment and soothing, (b) people pleasing by compliance and surrender; 4. Help seeking including (a) barriers to recovery from an eating disorder, (b) dissatisfaction with interventions experienced to date, (c) schema therapy as a promising alternative.

####  Adverse family environments

Most participants recognised their vulnerable child and felt that this had originated from various adverse family environments including instances of sexual abuse, bereavement, strict religious or military parents, neglect such as parental alcoholism, adoption, being sent to boarding school, having a mother with an ED and having siblings who had medical or learning disabilities that monopolised their parent’s attention. Upon exploration, participants were able to relate this to their vulnerable and angry child modes. Participants also discussed the contribution of maladaptive home environments to their need for control and the high level of demand that they placed on themselves in demanding parent and eating disorder overcontroller modes.

#####  Trauma - the vulnerable and angry child

Discussing the vulnerable child, participants shared the experience of vulnerability, abandonment, loneliness and feeling unworthy and unloved. For example, reflecting on her experience of trauma P4 shared:

*I grew up around a lot of child abuse… there were all sorts of other levels of dysfunction going on….it culminated in me being sent to boarding school and I was bullied the whole way through. I think there’s a lot of truth in the fact that you stop developing at the age of trauma. It’s something you can never leave behind ….so much damage and so much fracturing in childhood* (P4).

Other examples of participants acknowledging the negative impact of their upbringing in the development of their vulnerable child are below:

*We were bought up in a strict Christian household erm so it was quite negative….* (P6).

*My father was quite an angry person, very unpredictable. I never felt safe. I felt frightened almost all of the time. It was very shattering”* (P14).

*I was always sort of invisible coz he* {{a brother with learning difficulties}} *was always sort of a pain in the arse. Boarding school was not a brilliant experience for me. They used to call it character building, now they call it traumatizing* (P10).

Interestingly some participants did not score highly on vulnerable child, but during the interview recognised having experiences of vulnerability:

*I’ve a fear of abandonment of course. My mother used to abuse me …burn me and her dad was an alcoholic spent all his money on wine, women and song* (P12).

*My father was quite an angry person and I think quite depressed…….it was very rule orientated, lots of rules… I was criticised a lot. I never, I never felt, didn’t feel very safe, I felt, I felt frightened. My father was very unpredictable…… I wasn’t beaten but I was hit. Nobody stood up for me….mother didn’t say “hey,,,that’s not right* (P14).

Participants’ vulnerability was often accompanied by feelings of anger. They were able to relate this to having an angry child and pinpoint its origins as well as recognising that they switch between vulnerable child and angry child. For some the anger felt is a response to things that happened to them in childhood and their eating behaviours developed as a form of punishment:

*I’m just so angry that I can’t change the situation. Everything I say is almost denied…….and I think one way to express anger is through your body and its quite a giveaway and its harder for people to deny* (P1).

*Yeah, I didn’t have a brilliant childhood and was quite angry at my parents er they were alcoholics, so I struggled with that* (P2).

#####  Unrealistic standards – demand & control

Participants identified with the demanding mode again relating this back to early adverse family environments.

High academic expectations were considered by several participants as a source of the demanding mode:

*It was very much expected that you go to uni and erm you’re successful* (P6).

*I got a lot of positive feedback for doing well at school* (P7).

*There was a lot of academic pressure growing up, they sent me to a private school and if I didn’t get particular grades, I was kind of challenged on that* (P9).

*My dad had massively high standards, if I came home with 90% on a test, he’d quiz me on what the 10% I got wrong* (P10).

Demanding mode appeared to overlap with eating disorder overcontroller for many participants. Participants shared that they particularly identified with the questions on both these modes and gave accounts of feelings of the need to control their eating which formed part of their identity and was used as a way of achieving and regulating emotions in a manner they felt to be positive as the following quotes about eating disorder behaviours illustrate:

*I feel good again almost like I’ve achieved something like I’m back in control* (P6).

*Yeah, see that’s the only thing I can control is my eating* (P8).

*Oh yeah, my eating disorder is always triggered by moments in my life where I feel out of control……when I’m very restrictive of what I eat I feel like a success… When I do control what and how much I eat I feel settled I feel at peace I feel secure* (P9).

*It* {{control}} *is a release but at the same time makes everything worse* (P11).

####  Mode effects

Participants reported that their modes impacted both their everyday life and their eating disorder thoughts and behaviors.

#####  On everyday life

Several participants linked modes to behaviours and feelings in everyday life. Vulnerable child could create guilt (P6, P8), demanding mode led to unrealistic demands (P4), and demanding mode and eating disorders overcontroller led to perfectionism (P13, P14):

*I am very aware that I always feel like I’ve done something wrong even now as an adult and that’s like my default sort of try and prove that I’m ok aren’t I?* (P6).

*I feel a lot of guilt and that I let everybody down and that anything that might be wrong is my fault* (P8).

*Clearly my level of demand on myself is insane…. I mean it’s ridiculous it’s absolutely ridiculous it’s like the myth of Sisyphus the guy that spends all day every day pushing a massive boulder uphill and at the end of every day it falls down again and yet he does it again* (P4).

*I have to be perfect at everything. I have to be the perfect friend, I have to be the perfect girlfriend, I have to be the perfect student, the perfect daughter, sibling whatever you know? I can’t cope making mistakes* (P13).

*I’m tortured, I know, I know there’s a right and wrong way to do things….standards have to be lived up to….so that’s another issue as well because that perfectionism means that well, if I don’t try, I can’t get it wrong…if I don’t do something I haven’t got to live up to it….it’s another way of kind of snookering yourself…If I don’t do it I can’t get it wrong…but then that’s maladaptive because then nothing happens* (P14).

P14 indicates that perfectionism in demanding mode can be problematic as it can cause inaction and problematic thoughts and behaviours. She also said that she has learned to push herself less, which she felt to be positive. P6 felt similarly:

*Part of me feels ashamed of that because I’m not a highflyer like everyone else in my family but for me it’s safe and not in a negative way, I don’t think it’s a safety behaviour, I just don’t feel like I’m pushing myself in ways that are unhealthy* (P6).

#####  On disordered eating

Participants could also connect how the modes manifest in their thoughts and behaviours around their eating. For one participant, the feelings of abandonment and loneliness, bought on by the vulnerable child mode, manifested nowadays in her restrictive eating behaviours:

*The world makes no sense to me; people make no sense to me. I don’t feel like one of them. It’s very isolating. Restriction works, I’m a different person when fasting* (P10).

For others, family perceptions about their ED elicit their vulnerable child and then a coping mode. For P6, her troubled relationship with her sister in childhood continues to impact on her and elicit feelings of abandonment:

*My sister had eating disorders and was quite rebellious…. She started improving and got with this guy. I was really happy for her but I was having a particularly rough time and I just felt like she wasn’t there… I just felt abandoned….eating disorder behaviours became my little friend…I felt kind of safe and understood* (P6).

For P6 her ‘little friend’ is a form of detached protector mode. Others also described using ED as detached protector mode:

*I’ve got a very very strict father whose always been critical of my weight saying things like your putting on weight or you shouldn’t be eating that or you shouldn’t dress that way coz of your weight. I remember him always being like that with my mum. She left when I was fifteen….Yeah thanks you’ve left me in it now…* {{eating behaviours were a}} *kind of escape* (P11).

Another understands her eating is used as a suppression of anger *I’m quite ashamed of feeling angry. I often binge and puke actually when I’m feeling pissed off* (P6). This participant recognised that she switched from vulnerable child mode to angry child mode. After having her vulnerable child activated in relation to feeling abandoned by her sister for recovering from her ED and finding a new partner, she reports that during a conversation with her sister where a comment was made about her eating behaviours, she lost her temper *I just lost it…I went from being this mouse to just attacking her which is very out of character. The police were involved and everything* (P6).

Participants also reflected on how the demanding critic mode and how their disordered eating may have developed because of being unable to meet unrealistic demands.

*I got all the top grades but now I’m incapable of leaving the day if I’ve not done something to the standard necessary. I just don’t like not being the best I can be. If I’ve disappointed myself, if I stumble, I tend to eat badly* (P11).

####  Modes’ functions

Participants varied in which modes they scored highly on and recognised in themselves. Participants considered the results of these patterns of thoughts and behaviours, leading to two subthemes of emotion avoidance, generally with detached self-soother or detached protector, and people pleasing with compliant or helpless surrenderer.

#####  Emotion avoidance – detach & sooth

Participants used the detached self-soother coping mode as a distraction by engaging in behaviours such as excessive exercise, comfort eating and using alcohol to avoid or suppress difficult thoughts and emotions, as illustrated in the below quotes:

*I pace a lot, it kills time, it’s a really bad habit coz it also burns calories* (P1).

*Yeah, yeah just kind of bat them* {{emotions}} *away with food* (P2).

*I will do anything to avoid emotion…emotions hurt. I will anaesthetize myself at any opportunity* (P10).

*Yeah…. I’m almost a bit scared of big emotions erm I kind of like to talk myself out of things or do something to distract from big emotions. I really enjoy sport, running or swimming, it’s like an emotional release a way to kind of control my emotions* (P7).

While many recognised this was an unhealthy behaviour, several participants also indicated that it was effective as a way of calming anxiety and that they were fearful of giving up behaviours:

*If you’re keeping busy, the focus is away from your body and it’s onto something else* (P1).

*When my physical body is playing me up big time, I have to focus on something else* (P3).

Participants showed awareness of the detrimental effect of some of these coping behaviours on their eating disorder:

*I guess self-soothing with food is what I would say, and then of course that solution became the problem in itself because of binging….it’s very unpleasant…. physically and mentally…. I would spend my entire time thinking about eating or not eating…I mean to the detriment of everything else* (P14).

*I just felt drained and I just wanted to go and eat rubbish……to kind of cope, maybe I should’ve done it in a different way* (P2).

Participants also described using the detached protector mode, sometimes deliberately, to avoid being hurt by others. This appeared to emerge as a response to the vulnerable child with participants reflecting that although they felt lonely, vulnerable and feared abandonment, letting people close risked being let down and getting hurt, as well as having to address their eating disorder.

*I think emotional connection is really important but the eating disorder part of me feels just that people are a threat. I just feel a bit er toxic sometimes* (P6).

*It’s hard, people judge you, put a label on you and they see you differently as if it’s my fault, they do judge you and it’s really not helpful. So, I do it* {{detached protector}} *for sure I do it on purpose it’s a conscious decision* (P4).

*People hurt me….so I don’t allow the emotional connection. If I have nothing, then I don’t feel at risk because there’s nothing they can take away* (P10).

#####  People pleasing – compliance & surrender

When exploring relationships in their life, participants shared a common need to people please and avoid conflict whilst also feeling frustrated that others lack awareness of their needs. This was at odds with the need for protection from hurt as outlined above for detached protector and, as can be seen in Table 2, all but three participants had both a detached and a surrenderer mode.

In relation to the compliant surrenderer mode, participants voiced that it was just easier to let people have their own way. Furthermore, when asked why they do this it was explained that they feared people would not like or value them if they didn’t do what they wanted.

*I just try to be as easy as possible… I just want people to like me….I would hate it if somebody thought I was difficult* (P7).

*It’s acceptance it’s all down to acceptance* (P3).

*I can actually see myself doing it, just desperately trying to seek people’s approval* (P4).

Fear of confrontation was another element for several participants:

*My childhood growing up, I would never have stood my ground…….my mum, I feared her you know?* (P3).

*Yeah, I don’t like confrontation so I avoid it* (P8).

*If I felt there was going to be a confrontation, I would do anything to avoid it* (P10).

Again, as with the emotion avoidance behaviours, participants recognized that the compliant surrenderer can be counter-productive in that it could cause resentment due to feeling used by others:

*You’re giving them so much and not getting anything back and you feel you should be* (P2).

*I’m a bit of a people pleaser but then feel resentful coz I’m walked on even though I’ve chosen that path myself* (P6).

Many also identified with helpless surrenderer mode and participants identified the negatives of this. Some participants felt unable to change their behaviours, worried that if they recovered from their ED other people would not accept them, and recognised that their desire for people to understand them without the need for explanation was unrealistic:

*I like to think I can make changes…I try to make changes but its……you last like a week and then something happens* (P11).

*I can’t be bothered……you make a big effort and move on slightly it just gets sort of knocked out of me again…………I’m scared that people might not accept me in the future if I am a ‘sensible’ weight”* (P3).

*“I used to think that everybody knew but then I realised they don’t unless I actually spell it out* (P1).

*I mean I still don’t understand my eating behaviour and why I do what I do so I know its extra hard to expect other people to get it* (P6).

####  The desire for and the struggle to get help

Interviews focussed on modes, but all participants brought up the challenges of obtaining and working effectively with treatment. Two subthemes were barriers to recovery and dissatisfaction with previous treatment. None of the participants reported having engaged effectively with treatment for eating disorders and their general perspective was that they had not been offered treatment that they felt to be appropriate or effective. In this context, they felt that schema therapy was promising as an alternative.

#####  Barriers to recovery

Participants commented on the lack of support and understanding of their behaviours from family members, again reinforcing vulnerability:

*My dad’s a retired doctor and my sister is a consultant doctor; they have no time for the fluffy nonsense of mental health…they fix bodies. I often feel not good enough, like I’ve let them down or a disappointment* (P9).

*I kind of said to her you do realise mum that fatality with this condition is 20% and that I’ve been told I’m playing Russian Roulette with my heart, her answer was ‘Yeah but think about what I’ve been through’. Really mum? Really?!* (P4).

*There is never an acknowledgment* {{from their mother}}, *only that I am still a problem…defective…apparently, they are all absolutely fine….I think she’s perhaps got a bit of a problem with narcissism….makes it all about her …. if I broach this with her she simply goes quiet there is no discussion* (P14).

Participants also shared that they feared addressing their eating disorder due to an attachment to the behaviours and fearing a of loss of identity:

*Yeah, I often feel…it’s a bit warped…I do see my eating disorder as more problematic than I used to but I often feel a bit smug that I can do it and others can’t* (P6).

*It’s almost part of my identity which makes it harder to give up because who am I if I don’t look like this* (P1).

*It has a huge psychological effect on me it changes who I am as a person, and I prefer that person* (P10).

Participants were able to acknowledge an awareness of the downside of modes in respect of the recovery process, for example with the helpless surrenderer mode:

*Someone said to me once it’s not your fault, but it is your responsibility that was so helpful because it actually made me realise that it actually has to come down to me* (P1).

*You’ve got to take on that responsibility yourself because no one else can do it for you* (P3).

#####  Dissatisfaction with treatment

Despite not being the focus of the research, all participants shared that they had sought some form of help for their ED. Many expressed how frustrating accessing help is;

*They like to pretend eating disorders aren’t a problem really* (P11).

*It’s been impossible for me to get help because ‘You’re too complicated’* (P1).

*Erm, they tried to discharge me last week…they wanted to discharge me coz I’m too complex* (P4).

One participant shared that there is a lack of recognition of ED’s in older adults, and getting past their GP is a struggle:

*I was begging for help in the end. They want something they can fix….my GP admitted feeling helpless coz there was nothing he could do……Adult anorexics don’t exist, especially middle age ones* (P10).

Several participants expressed anger because interventions for EDs had involved an unhelpful focus on weight and food with some saying they felt patronised, belittled and not understood, seen or listened to:

*MY GOSH they are down your throat all the time* (P3).

*I think generally speaking, the help out there for people like me is shameful it’s actually doing more harm than good. Saying to me ‘you do understand that if you just put a bit of weight on then everything would be better?’ It’s such an insult. When are these people ever going to realise it’s got naff all to do with food. You need to solve the cause not the symptoms* (P4).

Participants discussed receiving CBT for comorbid anxiety and depression, with some specifically stating that they had not received CBT type interventions for their eating disorder. All felt that CBT was of limited usefulness:

*I have my CBT for everything else in my life but it’s not making me any better like it’s not working with the anorexia* (P9).

*I find CBT helps with trying to deal with it on a day-to-day basis but I’d like to know why I am this way coz I feel that would help figure out how to stop being this way* (P11).

Moreover, some felt CBT techniques did not transfer well beyond the clinic.

*It’s ok when you’re with the person at the time but taking it out into the real world the negative thoughts started to come back into my head and then carried on and you know I just ended up in another mess again…sort of a yo yo really* (P3).

Overall, participants reported dissatisfaction with previous attempts to offer treatment, that CBT type interventions had been unsuitable, and some remembered being labelled too complex for treatment to be effective.

#####  Schema therapy as an alternative

All participants felt that schema therapy had potential. They were positive about the approach for a variety of reasons such as:

*I am all for something that treats me, with respect, like an individual human being* (P4).

*it feels more individual* (P7).

*something that doesn’t put me in a box* (P8).

*I actually cried when I did the questionnaire………no no in quite a positive way in a sense because a lot of the questions were like Oh my God that’s how I feel* (P6).

Another participant felt that the CBT technique of seeking counter evidence for negative thoughts was of limited help:

*{{CBT}} Find evidence for and against a particular thought which is meant to help you….but I don’t really believe it coz I’m not really looking at what’s behind it. That’s where I think this {{schema therapy}} would be beneficial* (P6).

At the conclusion of the interviews, participants were asked if they had found the process beneficial. One participant stated in language reminiscent of schema theory:

*It makes a lotta sense…yeah…I can see where my past has affected me…….Listen you’re doing it coz you’re getting something out of it….its finding out what you’re getting out of it, what it is soothing, what is changing that’s making you feel better and most of that is tracked from our childhood that’s still kicking us in the head* (P10).

Another shared:

*I actually found it really useful because it had flagged those things….kind of…you know perhaps some things I was avoiding looking at, its kind of woke me up a little bit you know….I believe I was emotionally neglected and you know….you don’t realise that’s happened to you….I simply didn’t I didn’t get what I needed you know, you have a child in order to be a successful adult* (P14).

Finally, one participant who didn’t improve with CBT explained that private psychotherapy had helped her and drew an analogy with schema therapy:

*Yeah, I think one massive thing was she told me this isn’t about food, I’m not here to tell you how much to eat, I’m here to help you find out who you are. I found going on that journey very beneficial. Schema Therapy sounds similar and I think it sounds really interesting and enjoyable actually identifying different parts of yourself and balancing them out”* (P7).

## Discussion

Participants were engaged with an eating disorders charity. Compared to the stereotype of eating disorder clients mainly being young women, these women were older, and many had had their problems a long time. Nonetheless, they reported that they had not received extended specialist treatment, although it is likely that they had been offered, but had been unable to engage with such treatment. Common reasons for lack of engagement in England include a lack of specialist residential or community places and sometimes strict admission criteria, such as being below a specific body mass index. Participants in this study, whilst keen to recover, felt unable to engage in the treatments offered due to finding service practices unhelpful or patronising. Some also mentioned being too complex, which suggests that it had been recognised, at least informally, that CBT-E might not work well for them.

They shared common experiences of adverse childhood family environments, as found in previous research into schema modes and ED etiology [11, 29]. Most attributed their vulnerable child mode to such adversity. They also identified with the angry child, demanding parent and eating disorder overcontroller modes which often co-occurred with each other and tended to be activated once the vulnerable child was triggered. Participants talked about how these modes impacted on their everyday lives and their disordered eating. They also recognised the coping modes that they used in response to the activation of the above modes and realised the detrimental effects of these.

Participants discussed barriers to their desired recovery and adverse experiences of therapy. They reported that they felt that schema therapy had potential as an intervention suitable for them, whereas they had not been satisfied with previous cognitive behavioural therapy, which they tended to feel was simplistically about eating behaviours and insufficiently focussed on the underlying psychological causes of their eating disorder. Strikingly, despite no previous exposure to its concepts, participants felt comfortable discussing their experiences in the framework of schema therapy and engaged with the language in a positive manner.

Identification with the modes in this study supports findings from the quantitative studies reviewed above [15, 16, 19, 20] where the vulnerable child, demanding mode and detached protector, detached self-soother, helpless surrenderer and compliant surrenderer modes were found to be significantly higher in ED individuals across all ED subtypes. An interesting observation in the present study was that several participants qualitatively reported that they could recognise their vulnerable child despite not scoring 3 + on the questionnaire. Exploration of modes should not rely purely on questionnaires, but use clues in body language and dialogue [22].

Schema modes can switch rapidly [2], which was reported here and provides a framework to understanding different ED triggers. Participants recognised for example that activating vulnerable child could trigger angry child, and that demanding mode could trigger vulnerable child. Overcontroller mode is related to ED behaviours [20]. Here, demanding mode or eating disorders overcontroller could sometimes entirely override vulnerable child, leading to perfectionism as the only way to self-acceptance or acceptance by others [19]. When demanding mode was thwarted, then this could lead to eating disorders overcontroller, within that narrow domain which they could still control [2, 15]. As theorised by schema therapy, enduring eating disorders may be functional in that they can be controllable outcomes of mode switching. There may be a need to weaken other modes to prevent triggering eating disordered thoughts and behaviours. Notably, the person may need to learn to recognise and nurture their vulnerable child, by developing their healthy adult mode, rather than making perfectionist demands of themselves. Further qualitative research could focus on how modes switch and lead to disordered eating.

As found in past studies [11–14, 29, 30], relationships with those close to them were often complex and strained and many participants engaged in protective behaviour based on their past experiences to avoid being hurt further. They had developed the notion that others will hurt them and let them down and that they are the only person who they can rely on, keeping people at arm’s length; evident in the presence of the detached protector. Studies have shown that the detached protector is pronounced in the ED population compared to healthy controls and other groups such as those with obsessive compulsive disorder (OCD) or personality disorder and is particularly high in those with AN [15].

An unanticipated finding was participants sharing adverse experiences of therapy. All reported problems accessing appropriate treatment for their ED, for some because they had not met the specialist service’s criteria due to having BMI > 18. This led to further weight loss which was viewed as counterproductive, as in previous research [30]. Several participants also shared that they had been overlooked due to a lack of understanding or dismissed as too complex by GP’s and services. It has been suggested that some primary care staff perceive ED patients as difficult and requiring significant work due to the level of psychological and emotional support they require [31].

When CBT was received, it was recalled as being given for comorbid anxiety and depression. Participants felt that it could not address their ED. They felt that a behavioural focus on food, weight and exercise was insufficient, and that treatment should focus on personal issues and the psychological basis for these behaviours [32]. Most understood CBT principles, but felt that it did not address their long-standing core issues, which was frustrating [2, 5, 15, 17].

Participants consequently felt that schema therapy appeared to be a positive, alternative way of addressing their ED behaviours. They felt it could be a more personal and less prescriptive approach and that just talking through modes in the interview made sense and forced them to think about things differently and about things, such as their vulnerable child, that they were not generally aware of.

The cohort interviewed for this study was unusual in that they had enduring eating disorders which had often lasted a very long time. Eight of nine women with anorexia nervosa reported that the problem had lasted five years or more, whereas other types of eating disorder were under-represented. It is unclear that CBT-E is suitable for people with enduring anorexia nervosa, so it matters that this cohort felt that schema therapy might be beneficial for their enduring eating disorders and were able to identify with and discuss their condition using the technical language of schema modes.

## Data Availability

Due to the sensitivity of the qualitative data, participants only consented to share their data with the research team, so data are not publicly available.
